# Supplementary material for: A qualitative study of the barriers and enhancers to retention in care for pregnant and postpartum women living with HIV
Source: PLOS Glob Public Health. 2021 Oct 13;1(10):e0000004. doi: 10.1371/journal.pgph.0000004 (PMC10021710; doi:10.1371/journal.pgph.0000004)
Supplement: S1 Text — (DOCX) [file pgph.0000004.s001.docx]

# S1 Text. Interview guide for pregnant women, English version.

| **Enrollment Information** | |
| --- | --- |
| **Woman Study ID: ________**  **Date of interview: ________**  **Interviewer initials: ________** | **Location of Interview:**  □ Facility à name: __________________  □ Community à name: ______________ |

**Purpose Statement:**

To explore the barriers and facilitators to antenatal care (ANC) and Maternal and Child Health (MCH) attendance and adherence to HIV care for pregnant women with HIV.

**Key**:

à indicates potential probes and follow-up questions [*facility*] indicates study facility where subject enrolled in ANC

| **ANTENATAL QUESTON GUIDE FOR ALL PREGNANT WOMEN** | | |
| --- | --- | --- |
| **#** | **Topics** | **Question Guides** |
| **1.1** | General introduction | **Tell me about the community/town/village where you live.**  à **How long have you lived there?**  **How do you earn a living?**  à **Do you have other income generating activities?**  à **Do you own land / have access to land to cultivate? [If they have access to land, explore whether they cultivate only for subsistence or to sell]**  **Tell me about your household [explore whether they have electricity, how they cook food, how/where they get water]** |
| **1.2** | Challenges for pregnant women | **Tell me about the biggest challenges facing pregnant women in your community.**  **Tell me about the different types of care options that are available for pregnant women in your community.**  à **Ask specifically about each different care option in the community (e.g. traditional birth attendant, rural health center, pharmacy, dispensary, traditional healer/herbalist, district and/or sub-district health facility, and other care options)**  à **How does a woman decide which care option to use?**  à **What would you say is the most common?** |

|  |  | à **Why do you think that is the most common?** |
| --- | --- | --- |
| **1.3** | Perceptions of ANC in general | **Tell me why you decided to attend ANC for your pregnancy?**  **Tell me about the last time you heard someone in the community talking about ANC. What were they saying?**  à **What good things do people say about ANC?**  à **What bad things have you heard?** |
| **1.4** | Timing of ANC enrollment | **Why do some women first come to ANC early in pregnancy?**  **Why do some women wait until very late in their pregnancy to first come to ANC?**  **What do you think could be done to help women first come to ANC earlier in their pregnancies?** |
| **1.5** | Retention in ANC | **Why do some pregnant women keep their ANC appointments while others stop attending ANC?**  **What things help a woman to keep her ANC appointments?**  **What things might make it difficult for a woman to keep her ANC appointment?** |
| **1.6** | Barriers and facilitators to attending ANC | **What do you think are the major barriers for pregnant women coming to ANC clinic?**  **What does husband/partner think about you attending ANC?**  à **Is your husband/partner involved in your ANC care? In what ways?**  à **Are you satisfied with your husband/partner’s involvement in your ANC care now? Why or why not?**  **What does your mother think about you attending ANC?**  **What does your best friend think about you attending ANC?**  **What does your grandmother think about you attending ANC?**  **Give me some examples of the types of things that people in your community might say about pregnant women living with HIV?** |
| **1.7** | Personal experience concerning social perceptions of HIV and pregnancy | **Tell me about the last time you heard someone talking about a pregnant woman living with HIV?** |

|  |  | à **What were they saying?**  à **Who was talking?** |
| --- | --- | --- |
| **1.8** | Perceptions of ANC services at [*facility*] | **Tell me about your experience during your most recent visit to the ANC clinic at [*facility].***  à **What did you like about this experience?**  à **What did you not like about this experience?**  à **How could this experience be improved?**  à **What mode of transportation did you use? How much did it cost?**  à **Did anyone go with you?**  à **Tell me about how this appointment impacted your work/household/childcare responsibilities.** à **Did you tell your husband/partner about the appointment? Why or why not?**  **What do you think about the healthcare providers at the ANC clinic?**  à **What were the good characteristics about them?**  à **What were the bad characteristics about them?** |
| **1.9** | Perceptions about the integration of ANC and HIV services in the same clinic | **Tell me about your experience getting your HIV care in the ANC clinic?**  à **What did you like about this experience?**  à **What did you not like about this experience?**  à **How could this experience be improved?** |
| **1.10** | Recommendations for improving ANC attendance for pregnant women with HIV | **Specifically for pregnant women with HIV, what do you think would bring them into ANC earlier?**  **Specifically for pregnant women with HIV, what do you think would keep them coming to ANC after their first appointment?** |
| **1.11** | Challenges of managing HIV during pregnancy | **Tell me about the challenges pregnant women face when they are living with HIV.**  à **What are other challenges (repeated until no additional responses are elicited)**  à **Probe: What do you think would help them overcome these challenges?**  **Tell me about how a pregnant woman with HIV decides whether or not to take ART during their pregnancy.**  à **Why do you think some pregnant women with HIV do not take ART during their pregnancy?**  **What would help pregnant women with HIV continue taking HIV medications during pregnancy?**  **Tell me about the reasons a pregnant woman with HIV might NOT go to ANC.** |

|  |  | à **What are other reasons (repeated until no additional responses are elicited)** |
| --- | --- | --- |
| **1.12** | HIV disclosure | **What are some reasons a pregnant woman might not tell her husband/partner that she has HIV?** |

| **ADDITIONAL QUESTIONS FOR DISENGAGED PREGNANT WOMEN** | | |
| --- | --- | --- |
| **#** | **Topics** | **Questions** |
| **2.1** | Barriers and facilitators to attending ANC | **Tell me about why you have not returned to the clinic for so long.**  **What are some of the reasons why you have not gone to a clinic for HIV and ANC care?**  **How were you managing your HIV since you stopped coming to the [*facility*]?**  à **What has been easier for you?**  à **What has been harder for you?**  **What would help you go to receive ANC care in the next few weeks?**  **What challenges have you had in managing your HIV since your last visit to the clinic?** |

| **ADDITIONAL QUESTIONS FOR TRANSFERRED PREGNANT WOMEN** | | |
| --- | --- | --- |
| **#** | **Topics** | **Questions** |
| **3.1** | Reasons for transfer | **What made you decide to go to the new clinic for your ANC care?** |
| **3.2** | Perceptions about old and new ANC clinic | **What was your experience like at the new clinic?** à **What do you like about your new ANC clinic?** à **What do you dislike about your new ANC clinic?**  à **What did you not like about your old ANC clinic?** |

| **ADDITIONAL QUESTIONS FOR KNOWN POSITIVE WOMEN** | | |
| --- | --- | --- |
| **#** | **Topics** | **Questions** |
| **4.1** | Preferences for HIV care at the HIV clinic versus the ANC clinic | **In what ways is your experience receiving HIV care at your prior HIV clinic different than at the MCH clinic?** |

|  |  | **What do you like about your experience receiving your HIV care at the MCH clinic compared to your prior HIV clinic?**  **What do you not like about your experience receiving your HIV care at the MCH clinic compared to your prior HIV clinic?** |
| --- | --- | --- |

| **CLOSING QUESTIONS** |
| --- |
| **Is there anything else you think I should know?**  **Is there anything you would like to ask me?** |
| **Closing**: Thank participant for their time. Remind participant about the confidentiality of the interview. Ask if they have any questions about anything that was discussed. Offer information for care or other services and lunch/transportation allowance. |

# S1 Text. Interview guide for pregnant women, Kiswahili version.

| **Ujumbe wa uandikishaji** | |
| --- | --- |
| **Numbari ya utambulisho kwenywe utafiti:________________________**  **Tarehe ya mahojiano:_____________**  **Herufi ya mwanzo ya jina za mhojiwaji:_____________________** | **Eneo la mahojiano:**  □ Hospitali à Jina: __________________  □ Jamii à Jina: ______________ |

**Taarifa ya Kusudi:**

Kuchunguza vitu ambavyo vinazuia na kurahisisha kuenda kwenye huduma ya afya wakati wa ujauzito, afya ya mama na mtoto na kuzingatia huduma ya HIV kwa akina mama walio na HIV

**Ufunguo**:

à inaashira uwezekano wa maswali ya kufuatilia

[*Hospitali*] inaashiria hospitali ambayo mshiriki alijiandikisha kwa huduma ya afya wakati wa ujauzito

| **MWONGOZO WA MASWALI YA WAMAMA WOTE WAJAWAZITO** | | |
| --- | --- | --- |
| **#** | **Mada** | **Mwongozo wa maswali** |
| **1.1** | Utangulizi wa jumla | **Nieleze kuhusu jamii/mji/kijiji ambacho unaishi.**  à **Umeishi hapo kwa muda gani?**  **Unajimudu vipi kimaisha?**  à **Unanjia ingine ya kupata mapato?**  à **Je una miliki ardhi / una uwezo wa kupata shamba ya kulima? [Kama wana uwezo wa kupata shamba, uliza kama wanalima chakula chao cha kula ama cha kuuza]**  **Nieleze kuhusu nyambai kwako[uliza kama wana stima, jinsi wao hupika, ni aje ama ni wapi wao hupata maja]** |
| **1.2** | Changamoto za wamama wajawazito | **Nieleze kuhusu changamoto kuu zaidi zinazokumba wanawake wajawazito katika jamii yako.**  **Nieleze kuhusu aina tofauti za matibabu ambazo zinapatikana kwa wanawake wajawazito katika jamii yako.**  à **Uliza hasa kuhusu kila aina ya matibabu katika jamii (kwa mfano mkunga, hospitali ya kijijini, duka la dawa, dispensary, daktari wa kienyeji, hospitali ya wilaya na/ama hospitali ya wilaya ndogo, na huduma zinginezo ambazo zinapatikana)**  à **Ni vipi mwanamke huamua ni aina gani ya** |

|  |  | **huduma atatumia?**  à **Ni gani unaweza sema inatumika sana?**  à **Unadhani ni kwa nini ni inatumika sana?** |
| --- | --- | --- |
| **1.3** | Mtazamo wa ANC kwa jumla | **Nieleze kwa nini uliamua kuenda kwa ANC wakati wa ujauzito wako?**  **Nieleze kuhusu mara ya mwisho uliskia mtu katika jamii yako akizungumuza kuhusu ANC. Alikuwa anasema nini?**  à **Ni vitu gani mzuri watu husema kuhusu ANC?**  à **Ni vitu gani mbaya umeskia?** |
| **1.4** | Wakati wa kujisajili kwenye ANC | **Ni kwanini wanawake wengine huja kwenye ANC wakati mimba iko changa?**  **Ni kwanini wanawake wengine hungoja hadi siku za mwisho za ujauzito ndipo wanakuja kwenye ANC mara ya kwanza?**  **Unadhani ni nini inaweza fanywa kusaidia wanawake kukuja kwenye ANC mara ya kwanza wakati mimba yao ni changa?** |
| **1.5** | Uhifadhi kwenye ANC | **Ni kwa nini wanawake wengine wajawazito huja kwenye ANC wakati wa tarehe yao ya kliniki na wengine huwacha kuja kwenye kliniki ya ANC?**  **Ni vitu gani husaidia mwanamke kuja kwenywe ANC kwa ile tarehe ambayo ameandikiwa?**  **Ni vitu gani hufanya iwe ngumu kwa mwanamke kuja kwenywe ANC kwa ile tarehe ambayo ameandikiwa?** |
| **1.6** | Vitu vinavyozuia na kurahisisha wamama wajawazito kuhudhuria kliniki ya ujauzito | **Je, unafikiria ni baadhi ya vitu vipi vinavyo wazuia wamama wajawazito kuja cliniki ya ujauzito?**  **Je, mume/patna wako anafikiria nini kuhusu wewe kuhudhuria cliniki ya ujauzito?**  à **Mume/patna wako huhusika na kliniki zako za ujauzito? Anahusika kwa njia zipi?**  à **Umeridhika na kuhusika kwake na kliniki zako za ujauzito? Umeridhika/haujaridhika kwa sabau gani?**  **Je, mamako anafikiria nini kuhusu wewe kuhudhuria kliniki za ujauzito?**  **Je, rafiki yako wa karibu zaidi anafikiria nini kuhusu wewe kuhudhuria kliniki ya ujauzito?**  **Nyanyako naye anafikiria nini kuhusu wewe kuhudhuria kliniki ya ujauzito?**  **Nipe mifane ya baadhi ya vitu ambavyo watu katika** |

|  |  | **jamii yako husema kuhusu wanawake wanaoishi na virusi vya HIV ambao ni wajawazito?** |
| --- | --- | --- |
| **1.7** | Mambo yaliyoshuhudiwa kibinafsi kuhusu maoni ya jamii kuhusu HIV na ujauzito | **Nieleze kuhusu mara yako ya mwisho kusikia mtu akiongea kuhusu mwanamke mjamzito anayeishi na virusi vya HIV?**  à **Alikuwa anasema nini?**  à **Ni nani alikuwa anaongea?** |
| **1.8** | Maoni kuhusu huduma za ANC katika kituo cha afya cha [*jina*] | **Nieleze jinsi mambo yalivyokuwa ulipohudhuria kliniki yako ya mwisho ya ujauzito katika kituo cha afya cha [*facility].***  à **Ni nini ulipenda kuhusu jinsi mambo yalivyokuwa siku hiyo?**  à **Ni nini haukupenda kuhusu jinsi mambo yalivyokuwa siku hiyo?**  à **Ni vipi mambo kule katika kliniki yanaweza kuboreshwa?**  à **Ulisafiri namna gani kwenda kliniki? Ilikugharimu hela ngapi?**  à **Je, kuna mtu yeyote aliyeandamana nawe kwenda kliniki?**  à **Nieleze jinsi kliniki ya siku hiyo ilivyobadilisha majukumu yako kazini/nyumbani/ya ulezi.**  à **Je, ulimweleza mume/patna wako kuhusu kliniki ya siku hiyo? Sababu gani ilifanya umweleze/usimweleze?**  **Je, una maoni yapi kuhusu wale wahudumu wa afya katika kliniki ya ujauzito?**  à **Ni nini ilikuwa ya kupendeza kuwahusu?**  à **Ni nini haikuwa ya kupendeza kuwahusu?** |
| **1.9** | Maoni kuhusu kuwekwa pamoja kwa huduma za ANC na HIV katika kliniki moja. | **Nieleze kuhusu kupata matibabu ya HIV katika kliniki ya ujauzito?**  à **Ni nini ilikupendeza kuhusu kupata matibabu ya HIV katika kliniki ya ujauzito?**  à **Ni nini haikukupendeza kuhusu kupata matibabu ya HIV katika kliniki ya ujauzito?**  à **Hii hali inaweza kuboreshwa kwa njia gani?** |
| **1.10** | Mapendekezo kuhusu jinsi ya kuwafanya wanawake wajawazito wanaoishi na HIV kuhudhuria kliniki ya ANC zaidi | **Kwa wanawake wajawazito wanaoishi na virusi vya HIV, ni nini unafikiria itaweza kuwafanya waje kliniki ya ujauzito mapema?**  **Kwa wanawake wajawazito wanaoishi na virusi vya HIV, ni nini unafikiria itaweza kuwafanya kuja kilamara katika kliniki ya ujauzito baada ya kuja mara ya kwanza?** |
| **1.11** | Changamoto za kukabiliana na HIV wakati wa ujauzito | **Nieleze changamoto ambazo wanawake wajawazito hupitia iwapo wanaishi na virusi vya HIV.**  à **Ni changamoto zipi zingine? (Rudia hadi majibu yaishe)**  à **Uliza: Ni nini unafikiria itawasaidia kukabiliana** |

|  |  | **na hizi changamoto?**  **Nieleze jinsi mwanamke mjamzito anayeishi na virusi vya HIV huamua kama ataendelea kutumia madawa ya ART wakati wa ujauzito au la.**  à **Unafikiria ni kwa nini wanawake wajawazito wanaoishi na HIV hawatumii madawa ya ART wakati wa ujauzito wao?**  **Ni nini itawasaidia wanawake wajawazito wanaoishi na HIV kuendelea kutumia madawa ya HIV wakati wa ujauzito?**  **Nieleze kuhusu sababu ambazo huenda zikamfanya mwanamke mjamzito anayeishi na HIV asiende katika kliniki ya ujauzito.**  à **Ni sababu zipi zingine? (Rudia hadi majibu yaishe)** |
| --- | --- | --- |
| **1.12** | Kueleza wengine kuhusu kuwa na HIV | **Je, ni sababu gani zinaweza kumfanya mwanamke mjamzito aliye na HIV kutomwambia mume/patna wake kwamba ana virusi vya HIV?** |

| **MASWALI MENGINE YA WAMAMA WAJAWAZITO AMBAO WAMEACHA KUJA KLINIKI** | | |
| --- | --- | --- |
| **#** | **Mada** | **Maswali** |
| **2.1** | Vitu ambavyo vinazuia na kurahisisha kukuja kiliniki kwa wamama wajawazito | **Nieleze kuhusu wewe kutokuja kliniki kwa muda mrefu**  **Ni baadhi ya sababu gani zimekusababisha kutoenda kliniki ya HIV na ya wamama wajawazito?**  **Unakabiliana aje na hali yako ya HIV tangu uache kukuja hospitalini**  à**Ni nini imekuwa rahisi kwako?**  à**Ni nini imekuwa ngumu kwako?**  **Ni nini itakuwezesha kwenda kliniki ya wamama wajawazito hivi karibuni?**  **Ni changamoto gani umekuwa nazo ukikabiliana na hali yako ya HIV tangu mara yako ya mwisho kuenda kliniki?** |

| **MASWALI MENGINE YA WAMAMA WAJAWAZITO AMBAO WAMEBADILISHA KLINIKI** | | |
| --- | --- | --- |
| **#** | **Mada** | **Maswali** |
| **3.1** | Sababu za kubadilisha kliniki | **Nini ilisababisha uamuzi wako wa kubadilisha kliniki ya wamama wajawazito?** |

| **3.2** | Maoni kuhusu kliniki ya kitambo na ya | **Ni vitu gani umepitia katika kliniki yako mpya ya** |
| --- | --- | --- |
|  | saa hii ya wamama wajawazito | **wamama wajawazito?** |
|  |  | à **Unapenda nini katika kliniki yako mpya ya** |
|  |  | **wamama wajawazito?** |
|  |  | à **Ni nini hupendi kwa kliniki yako mpya ya** |
|  |  | **wamama wajawazito?** |
|  |  | à **Ni nini hukupenda katika kliniki yako ya kitambo** |
|  |  | **ya wamama wajawazito?** |

| **ADDITIONAL QUESTIONS FOR KNOWN POSITIVE WOMEN** | | |
| --- | --- | --- |
| **#** | **Mada** | **Maswali** |
| **4.1** | Preferences for HIV care at the HIV clinic versus the ANC clinic | **Kuna tofauti gani baina ya huduma uliyokua ukipata kwa kliniki yako ya virusi/H.I.V ya zamani na unayopata sasa kwa kliniki ya mama waja wazito/MCH?**  **Ni jambo gani limekupendeza kuhusu huduma za virusi vya H.I.V unayopata katika kliniki ya waja wazito/MCH ukilinganisha na kliniki yako ya**  **H.I.V ya zamani?**  **Ni jambo gani halikupendezi kuhusu huduma za virusi vya H.I.V unayopata katika kliniki ya waja wazito/H.I.V ukilinganisha na kliniki yako ya H.I.V ya zamani?** |

| **MASWALI YA KUMALIZIA** |
| --- |
| **Kuna jambo lolote ungependa kunielezea?**  **Kuna kitu chochote ungependa kuniuliza?** |
| **Kumaliza**: Mshukuru mshiriki kwa muda wao. Mkumbushe mshiriki juu ya usiri wa mahojiano. Uliza kama ako na swali kuhusu kitu chochote ilichojadiliwa. Peana habari kuhusu huduma ya afya na pesa ya mlo au nauli |
