# Supplementary material for: A qualitative study of the barriers and enhancers to retention in care for pregnant and postpartum women living with HIV
Source: PLOS Glob Public Health. 2021 Oct 13;1(10):e0000004. doi: 10.1371/journal.pgph.0000004 (PMC10021710; doi:10.1371/journal.pgph.0000004)
Supplement: S2 Text — (DOCX) [file pgph.0000004.s002.docx]

# S2 Text. Interview guide for postpartum women, English versions.

| **Enrollment Information** | |
| --- | --- |
| **Woman Study ID: ________**  **Date of interview: ________**  **Interviewer initials: ________** | **Location of Interview:**  □ Facility à name: __________________  □ Community à name: ______________ |

**Purpose Statement:**

To explore the barriers and facilitators to antenatal care (ANC) and Maternal and Child Health (MCH) attendance and adherence to HIV care for pregnant women with HIV.

**Key**:

à indicates potential probes and follow-up questions [*facility*] indicates study facility where subject enrolled in ANC

| **ANTENATAL QUESTON GUIDE FOR ALL POSTPARTUM WOMEN** | | |
| --- | --- | --- |
| **#** | **Topics** | **Question Guides** |
| **1.1** | General introduction | **Tell me about the community/town/village where you live.**  à **How long have you lived there?**  **How do you earn a living?**  à **Do you have other income generating activities?**  à **Do you own land / have access to land to cultivate? [If they have access to land, explore whether they cultivate only for subsistence or to sell]**  **Tell me about your household [explore whether they have electricity, how they cook food, how/where they get water]** |
| **1.2** | Challenges for postpartum women | **Tell me about the biggest challenges facing women who have recently had a baby in your community.**  **Tell me about the different types of care options that are available for women who have recently had a baby in your community.**  à **Ask specifically about each different care option in the community (e.g. traditional birth attendant, rural health center, pharmacy, dispensary, traditional healer/herbalist, district and/or sub-district health facility, and other care options)**  à **How does a woman decide which care option to use?** |

|  |  | à **What would you say is the most common?**  à **Why do you think that is the most common?** |
| --- | --- | --- |
| **1.3** | Perceptions of MCH in general | **Tell me why you decided to attend MCH after delivery?**  **Tell me why you decided to bring your baby to MCH after delivery?**  **Tell me about the last time you heard someone in the community talking about MCH. What were they saying?**  à **What good things do people say about MCH?**  à **What bad things have you heard?** |
| **1.4** | Timing of MCH enrollment | **Why do some women first come to MCH with their baby soon after they have delivered?**  **Why do some women wait until very late after they have delivered to come with their baby to MCH?**  **What do you think could be done to help women first come to MCH with their babies sooner after delivery?** |
| **1.5** | Retention in MCH | **Why do some women keep their MCH appointments while others stop attending MCH?**  **What things help a woman to keep her MCH appointments?**  **What things might make it difficult for a woman to keep her MCH appointment?** |
| **1.6** | Barriers and facilitators to attending MCH | **What do you think are the major barriers for women coming to MCH clinic with their babies?**  **What does your husband/partner think about you attending MCH with your baby?**  à **Is your husband/partner involved in your MCH care? In what ways?**  à **Are you satisfied with your husband/partner’s involvement in your MCH care now? Why or why not?**  **What does your mother think about you attending MCH with your baby?**  **What does your best friend think about you attending MCH with your baby?**  **What does your grandmother think about you attending MCH with your baby?** |

|  |  | **Give me some examples of the types of things that people in your community might say about women living with HIV after they have had a baby?** |
| --- | --- | --- |
| **1.7** | Personal experience concerning social perceptions of HIV after delivery | **Tell me about the last time you heard someone talking about a woman living with HIV after she has had a baby?**  à **What were they saying?**  à **Who was talking?** |
| **1.8** | Perceptions of MCH services at [*facility*] | **Tell me about your experience during your most recent visit to the MCH clinic at [*facility].***  à **What did you like about this experience?**  à **What did you not like about this experience?**  à **How could this experience be improved?**  à **What mode of transportation did you use? How much did it cost?**  à **Did anyone go with you?**  à **Tell me about how this appointment impacted your work/household/childcare responsibilities.** à **Did you tell your husband/partner about the appointment? Why or why not?**  **What do you think about the healthcare providers at the MCH clinic?**  à **What were the good characteristics about them?**  à **What were the bad characteristics about them?** |
| **1.9** | Perceptions about the integration of MCH and HIV services in the same clinic | **Tell me about your experience getting your HIV care in the MCH clinic?**  à **What did you like about this experience?**  à **What did you not like about this experience?**  à **How could this experience be improved?**  **Tell me about your experience getting care for your HIV-exposed baby in the MCH clinic?**  à **What did you like about this experience?**  à **What did you not like about this experience?**  à **How could this experience be improved?** |
| **1.10** | Recommendations for improving MCH attendance for women with HIV and their HIV-exposed infants | **Specifically for women living with HIV and their HIV-exposed babies, what do you think would bring them into MCH earlier?**  **Specifically for women living with HIV and their HIV-exposed babies, what do you think would keep them coming to MCH after their first appointment?** |
| **1.11** | Challenges of managing HIV after delivery | **Tell me about the challenges women face when they are living with HIV after they have recently had a baby.**  à **What are other challenges (repeated until no additional responses are elicited)** |

|  |  | à **Probe: What do you think would help them overcome these challenges?**  **Tell me about how a woman with HIV decides whether or not to take ART after she has had a baby.**  à **Why do you think some women with HIV do not take ART they have delivered their baby?**  **Tell me about how a woman with HIV decides whether or not to give ARV prophylaxis to their baby.**  à **Why do you think some women with HIV do not give ARV prophylaxis to their baby?**  **What would help women with HIV continue taking HIV medications after they have delivered their baby?**  **What would help women with HIV continue giving ARV prophylaxis to their baby after delivery?**  **Tell me about the reasons a woman with HIV might NOT go to MCH with her baby.**  à **What are other reasons (repeated until no additional responses are elicited)** |
| --- | --- | --- |
| **1.12** | HIV disclosure | **What are some reasons a woman might not tell her husband/partner that she has HIV?** |

| **ADDITIONAL QUESTIONS FOR DISENGAGED WOMEN-INFANT PAIRS** | | |
| --- | --- | --- |
| **#** | **Topics** | **Questions** |
| **2.1** | Barriers and facilitators to attending MCH | **Tell me about why you have not returned to the clinic with your baby for so long.**  **What are some of the reasons why you have not gone to a clinic for HIV and MCH care?**  **What are some of the reasons why you have not brought your baby for MCH care?**  **How were you managing your HIV since you stopped coming to the [*facility*]?**  à **What has been easier for you?**  à **What has been harder for you?**  **What would help you go to receive MCH care for yourself and your baby in the next few weeks?** |

|  |  | **What challenges have you had in managing your HIV since your last visit to the clinic?**  **What challenges have you had in managing your baby since your last visit to the clinic?** |
| --- | --- | --- |

| **ADDITIONAL QUESTIONS FOR TRANSFERRED POSTPARTUM WOMEN** | | |
| --- | --- | --- |
| **#** | **Topics** | **Questions** |
| **3.1** | Reasons for transfer | **What made you decide to go to the new MCH clinic for your care and for your baby’s care?** |
| **3.2** | Perceptions about old and new ANC | **What was your experience like at the new MCH** |
|  | clinic | **clinic?** |
|  |  | à **What do you like about your new MCH clinic?** |
|  |  | à **What do you dislike about your new MCH** |
|  |  | **clinic?** |
|  |  | à **What did you not like about your old MCH** |
|  |  | **clinic?** |

| **ADDITIONAL QUESTIONS FOR KNOWN POSITIVE WOMEN** | | |
| --- | --- | --- |
| **#** | **Topics** | **Questions** |
| **4.1** | Preferences for HIV care at the HIV clinic versus the ANC clinic | **In what ways is your experience receiving HIV care at your prior HIV clinic different than at the MCH clinic?**  **What do you like about your experience receiving your HIV care at the MCH clinic compared to your prior HIV clinic?**  **What do you not like about your experience receiving your HIV care at the MCH clinic compared to your prior HIV clinic?** |

| **CLOSING QUESTIONS** |
| --- |
| **Is there anything else you think I should know?**  **Is there anything you would like to ask me?** |
| **Closing**: Thank participant for their time. Remind participant about the confidentiality of the interview. Ask if they have any questions about anything that was discussed. Offer information for care or other services and lunch/transportation allowance. |

# S2 Text. Interview guide for postpartum women, Kiswahili version.

| **Ujumbe wa uandikishaji** | |
| --- | --- |
| **Numbari ya utambulisho kwenywe utafiti:________________________**  **Tarehe ya mahojiano:_____________**  **Herufi ya mwanzo ya jina za mhojiwaji:_____________________** | **Eneo la mahojiano:**  □ Hospitali à Jina: __________________  □ Jamii à Jina: ______________ |

**Taarifa ya Kusudi:**

Kuchunguza vitu ambavyo vinazuia na kurahisisha kuenda kwenye huduma ya afya wakati wa ujauzito, afya ya mama na mtoto na kuzingatia huduma ya HIV kwa akina mama walio na HIV

**Ufunguo**:

à inaashira uwezekano wa maswali ya kufuatilia

[*Hospitali*] inaashiria hospitali ambayo mshiriki alijiandikisha kwa huduma ya afya wakati wa ujauzito

| **MWONGOZO WA MASWALI YA WAMAMA WOTE WAJAWAZITO** | | |
| --- | --- | --- |
| **#** | **Mada** | **Mwongozo wa maswali** |
| **1.1** | Utangulizi wa jumla | **Nieleze kuhusu jamii/mji/kijiji ambacho unaishi.**  à **Umeishi hapo kwa muda gani?**  **Unajimudu vipi kimaisha?**  à **Unanjia ingine ya kupata mapato?**  à **Je una miliki ardhi / una uwezo wa kupata shamba ya kulima? [Kama wana uwezo wa kupata shamba, uliza kama wanalima chakula chao cha kula ama cha kuuza]**  **Nieleze kuhusu nyambai kwako[uliza kama wana stima, jinsi wao hupika, ni aje ama ni wapi wao hupata maja]** |
| **1.2** | Changamoto za wamama baada ya kujifungua | **Nieleze kuhusu changamoto kuu zaidi zinazokumba wanawake ambao wamepata watoto hivi majuzi katika jamii yako.**  **Nieleze kuhusu aina tofauti za matibabu ambazo zinapatikana kwa wanawake ambao wamepata watoto hivi majuzi katika jamii yako.**  à **Uliza hasa kuhusu kila aina ya matibabu katika jamii (kwa mfano mkunga, hospitali ya kijijini, duka la dawa, dispensary, daktari wa kienyeji, hospitali ya wilaya na/ama hospitali ya wilaya ndogo, na huduma zinginezo ambazo zinapatikana)**  à **Ni vipi mwanamke huamua ni aina gani ya** |

|  |  | **huduma atatumia?**  à **Ni gani unaweza sema inatumika sana?**  à **Unadhani ni kwa nini ni inatumika sana?** |
| --- | --- | --- |
| **1.3** | Mtazamo wa MCH kwa jumla | **Nieleze kwa nini uliamua kuenda kwa MCH baada ya kujifungua?**  **Nieleze kwa nini uliamua kumuleta mtoto wako kwenye MCH baada ya kujifungua?**  **Nieleze kuhusu mara ya mwisho uliskia mtu katika jamii yako akizungumuza kuhusu MCH. Alikuwa anasema nini?**  à **Ni vitu gani mzuri watu husema kuhusu MCH?**  à **Ni vitu gani mbaya umeskia?** |
| **1.4** | Wakati wa kujisajili kwenye MCH | **Ni kwanini wanawake wengine huja na wanao kwenye MCH punde tu baada ya kujifungua?**  **Ni kwanini wanawake wengine hungoja kwa muda mrefu baada ya kujifungua ndipo wanakuja kwenye MCH?**  **Unadhani ni nini inaweza fanywa kusaidia wanawake kukuja kwenye MCH na watoto wao punde tu wanapojifungua?** |
| **1.5** | Uhifadhi kwenye MCH | **Ni kwa nini wanawake wengine huja kwenye MCH wakati wa tarehe yao ya kliniki na wengine huwacha kuja kwenye kliniki ya MCH?**  **Ni vitu gani husaidia mwanamke kuja kwenywe MCH kwa ile tarehe ambayo ameandikiwa?**  **Ni vitu gani hufanya iwe ngumu kwa mwanamke kuja kwenywe MCH kwa ile tarehe ambayo ameandikiwa?** |
| **1.6** | Vitu vinavyozuia na kurahisisha wamama kuhudhuria MCH | **Je, unafikiria ni baadhi ya vitu vipi vinavyowazuia kinamama kuja MCH na watoto wao?**  **Je, mume/patna wako anafikiria nini kuhusu wewe kuhudhuria MCH na mtoto wako?**  à **Mume/patna wako huwa anahusika na huduma zako za MCH? Anahusika kwa njia zipi?**  à **Umeridhika na kuhusika kwa mume/patna wako kwa wakati huu na wewe kuhudumiwa MCH?**  **Umeridhika/haujaridhika kwa sabau gani?**  **Je, mamako anafikiria nini kuhusu wewe kuhudhuria MCH na mtoto wako?**  **Je, rafiki yako wa karibu zaidi anafikiria nini kuhusu wewe kuhudhuria MCH na mtoto wako?** |

|  |  | **Je, nyanyako naye anafikiria nini kuhusu wewe kuhudhuria MCH na mtoto wako?**  **Nipe mifane ya baadhi ya vitu ambavyo watu katika jamii yako husema kuhusu wanawake wanaoishi na virusi vya HIV baada ya hao kujifungua?** |
| --- | --- | --- |
| **1.7** | Mambo yaliyoshuhudiwa kibinafsi kuhusu maoni ya jamii kuhusu HIV baada ya kujifungua | **Nieleze kuhusu mara yako ya mwisho kusikia mtu akiongea kuhusu mwanamke anayeishi na HIV baada ya yeye kujifungua?**  à **Alikuwa anasema nini?**  à **Ni nani alikuwa anaongea?** |
| **1.8** | Maoni kuhusu huduma za MCH katika kituo cha afya cha [*jina*] | **Nieleze kuhusu mambo uliyoshuhudia ulipohudhuria kliniki yako ya mwisho ya MCH katika kituo cha afya cha [*jina la kituo cha afya].*** à **Ni nini ulipenda kuhusu mambo uliyoyashuhudia?**  à **Ni nini haukupenda kuhusu mambo uliyoyashuhudia?**  à **Ni vipi mambo katika kliniki yanaweza kuboreshwa?**  à **Ulisafiri namna gani kwenda kliniki? Ilikugharimu hela ngapi?**  à **Je, kuna mtu yeyote aliyeandamana nawe kwenda kliniki?**  à **Nieleze jinsi kliniki ya siku hiyo ilivyobadilisha majukumu yako kazini/nyumbani/ya ulezi.**  à **Je, ulimweleza mume/patna wako kuhusu kliniki yako ya siku hiyo? Sababu gani ilifanya umweleze/usimweleze?**  **Je, una maoni yapi kuhusu wale wahudumu wa afya wa MCH?**  à **Ni nini ilikuwa ya kupendeza kuwahusu?**  à **Ni nini haikuwa ya kupendeza kuwahusu?** |
| **1.9** | Maoni kuhusu kuwekwa pamoja kwa huduma za MCH na HIV katika kliniki moja | **Nieleze kuhusu kupata kwako matibabu ya HIV katika kliniki ya MCH?**  à **Ni nini ilikupendeza kuhusu kupata matibabu ya HIV katika kliniki ya MCH?**  à **Ni nini haikukupendeza kuhusu kupata matibabu ya HIV katika kliniki ya MCH?**  à **Hii hali inaweza kuboreshwa kwa njia gani?** |
| **1.10** | Mapendekezo kuhusu jinsi ya kuwafanya wamama wanaoishi na HIV pamoja na watoto wao ambao wako kwenye hatari ya kuambukizwa na HIV kuhudhuria kliniki ya MCH zaidi | **Kwa wanawake wajawazito wanaoishi na virusi vya HIV pamoja na watoto wao ambao wako hatarini kuambukizwa na HIV, ni nini unafikiria itaweza kuwafanya waje kliniki ya MCH mapema?**  **Kwa wanawake wajawazito wanaoishi na virusi vya HIV pamoja na watoto wao walio hatarini kuambukizwa na HIV, ni nini unafikiria itaweza kuwafanya kuja kilamara katika kliniki ya MCH baada ya kuja mara ya kwanza?** |

| **1.11** | Changamoto za kukabiliana na HIV baada ya kujifungua | **Nieleze changamoto ambazo wanawake hupitia iwapo wanaishi na virusi vya HIV na wamepata mtoto.**  à **Ni changamoto zipi zingine? (Rudia hadi majibu yaishe)**  à **Uliza: Ni nini unafikiria itawasaidia kukabiliana na hizi changamoto?**  **Nieleze jinsi mwanamke anayeishi na HIV huamua kama ataendelea kutumia madawa ya ART baada ya kujifungua au la.**  à **Unafikiria ni kwa nini wanawake wanaoishi na HIV hawatumii madawa ya ART baada ya kujifungua?**  **Ni nini itawasaidia wanawake wanaoishi na HIV kuendelea kutumia madawa ya HIV baada ya kujifungua?**  **Ni nini itawasaidia wanawake wanaoishi na HIV kuendelea kuwapa watoto wao matibabu ya ARVs baada ya kujifungua?**  **Nieleze kuhusu sababu ambazo huenda zikamfanya mwanamke anayeishi na HIV asiende na mtoto wake katika kliniki ya MCH.**  à **Ni sababu zipi zingine? (Rudia hadi majibu yaishe)** |
| --- | --- | --- |
| **1.12** | Kueleza wengine kuhusu kuwa na HIV | **Je, ni sababu gani zinaweza kumfanya mwanamke kutomwambia mume/patna wake kwamba ana virusi vya HIV?** |

| MASWALI MENGINE YA WAMAMA WALIO NA WATOTO | | |
| --- | --- | --- |
| **#** | **Mada** | **Maswali** |
| **2.1** | Vitu ambavyo vinazuia na kurahisisha kukuja kiliniki kwa MCH | **Nieleze kuhusu wewe kutokuja kliniki na mtoto kwa muda mrefu**  **Ni baadhi ya sababu gani zimekusababisha kutoenda kliniki ya HIV na ya MCH**  **Ni baadhi ya sababu gani zimekusababisha kutopeleka mtoto kliniki?**  **Unakabiliana aje na hali yako ya HIV tangu uache kukuja hospitalini?**  à **Ni nini imekuwa rahisi kwako?**  à **Ni nini imekuwa ngumu kwako?**  **Ni nini itakuwezesha kwenda kliniki ya MCH ili wewe na mtoto mhudumiwe hivi karibuni?** |

**MASWALI YA KUMALIZIA**

|  |  | **Umepitia changamoto gani ukikabiliana na hali yako ya HIV tangu mara yako ya mwisho kuenda kliniki?**  **Umepitia changamoto gani unaposhughulikia mtoto wako tangu mara yako ya mwisho kuenda kliniki?** |
| --- | --- | --- |

| **MASWALI MENGINE YA WAMAMA WALIOJIFUNGUA AMBAO WAMEBADILISHA KLINIKI** | | |
| --- | --- | --- |
| **#** | **Mada** | **Maswali** |
| **3.1** | Sababu za kubadilisha kliniki | **Nini ilisababisha uamuzi wako wa kubadilisha kliniki ya wamama walio na watoto ili uhudumiwe na mtoto wako?** |
| **3.2** | Maoni kuhusu kliniki ya kitambo na ya saa hii ya wamama walio na watoto | **Ni vitu gani umepitia katika kliniki yako mpya ya MCH?**  à **Unapenda nini katika kliniki yako mpya ya MCH?**  à **Ni nini hupendi katika kliniki yako mpya ya MCH?**  à **Ni nini hukupenda katika kliniki yako ya kitambo ya MCH?** |

| **ADDITIONAL QUESTIONS FOR KNOWN POSITIVE WOMEN** | | |
| --- | --- | --- |
| **#** | **Mada** | **Maswali** |
| **4.1** | Preferences for HIV care at the HIV clinic versus the ANC clinic | **Kuna tofauti gani baina ya huduma uliyokua ukipata kwa kliniki yako ya virusi/H.I.V ya zamani na unayopata sasa kwa kliniki ya mama waja wazito/MCH?**  **Ni jambo gani limekupendeza kuhusu huduma za virusi vya H.I.V unayopata katika kliniki ya waja wazito/MCH ukilinganisha na kliniki yako ya**  **H.I.V ya zamani?**  **Ni jambo gani halikupendezi kuhusu huduma za virusi vya H.I.V unayopata katika kliniki ya waja wazito/H.I.V ukilinganisha na kliniki yako ya H.I.V ya zamani?** |

**Kuna jambo lolote ungependa kunielezea?**

**Kuna kitu chochote ungependa kuniuliza?**

**Kumaliza**: Mshukuru mshiriki kwa muda wake. Mkumbushe mshiriki juu ya usiri wa mahojiano. Uliza kama ako na swali kuhusu kitu chochote ilichojadiliwa. Peana habari kuhusu huduma ya afya na pesa ya mlo au nauli
