# Supplementary material for: A qualitative study of the barriers and enhancers to retention in care for pregnant and postpartum women living with HIV
Source: PLOS Glob Public Health. 2021 Oct 13;1(10):e0000004. doi: 10.1371/journal.pgph.0000004 (PMC10021710; doi:10.1371/journal.pgph.0000004)
Supplement: Attachment — Submitted filename: Response to reviewers.docx [file pgph.0000004.s003.docx]

**Response to Reviewers**

Key:

bold text = reviewer’s comment

plain text = authors’ response

underlined text = text added to revised manuscript

**Reviewer #1**

1. **Considering the fact that LTFU women have an opportunity to voice the reasons that hampered for them, and their children, access to health and HIV services, authors should justify what is the rationale for offering in parallel ‘second-hand’ perceptions of women retained in care about LTFU ones.**

We have added the following text to the Introduction:

“Finally, most studies assessed factors influencing retention only among PPHIV who were retained in care. Understanding these factors according to retained PPHIV is important because these women may have experienced, and successfully overcome, a variety of barriers to retention, giving them unique perspectives on the relative importance of each factor. However, by not sampling PPHIV who had become LTFU, these studies may not have captured the full range of factors influencing retention in care for this population [31].”

2. **The Discussion (and consequently conclusion) would benefit from further refection on how integrated PMTCT services, at the sites were participants in the study were recruited, are organized and where potential changes/ improvements can be introduced to minimize lost to follow up.**

We have included the following text to the Discussion:

“However, several structural barriers within the healthcare environment suggest opportunities for interventions. For example, retained and LTFU women perceived that integrated services increased the risk of HIV status disclosure. All of the facilities in our study had a common reception area and separate clinical areas for HIV-positive and negative women, respectively. Thus, it is possible that patients could identify one another’s HIV status based on their triage path within the clinic. Reconfiguring these clinics to better protect patient privacy by using private clinic rooms that serve both HIV positive and negative clients could enhance retention for some PPHIV. This may not be feasible at some clinics, however. The ANC and PNC sites used for recruitment in our study were nested within public referral facilities and had limited space for triage, patient encounters, recordkeeping, and other ancillary services which would not be easily reconfigured to enhance patient privacy. This highlights a major challenge of integration in resource-constrained clinics, which may not have sufficient capacity to handle the additional service needs of many PPHIV in high HIV burden settings. To reduce the risk of attrition, some women may be or offering women a choice to receive integrated or non-integrated services, particularly if their partner is also living with HIV, which may also help to promote male engagement in PMTCT and family-centered care.”

“Differentiated service delivery models for PPHIV are lacking, however, and research is needed to understand the adaptation of this model to the PMTCT population [41, 53, 54]. These models may also help reduce the risk of stigma and disclosure at integrated clinics by lessening appointment frequencies and durations for PPHIV, aligning them more closely with HIV negative women and minimizing these elements as distinguishing characteristics between both groups [30].”

We have also included the following text in the Conclusion:

“HIV care programs offering integrated HIV and maternal and child health services should also carefully assess the configuration of the clinics in which integrated services are being delivered, and the ways in which it may enhance or diminish retention in care for PPHIV. These issues represent critical challenge for care programs and policy organizations given the magnitude of non-retention in the PMTCT continuum in sub-Saharan Africa.”

**Reviewer #2**

1. **In the introduction the authors write that “many studies were not conducted in settings in which HIV services were integrated in maternal and child health clinics. These settings may present unique barriers to retention compared to non-integrated settings”. Can you specify and elaborate on these barriers? This would be useful to further highlight the value of the paper.**

We have added the following text to the Introduction:

“Furthermore, many studies were not conducted in settings in which HIV services were integrated in maternal and child health clinics. These settings may present unique barriers to retention compared to non-integrated settings [26-29]. Such barriers include heightened stigma and fear of public disclosure at clinics providing care to women living with and without HIV, and increased provider workload increasing patient wait times and impacting patient-provider relationships [30].”

2. **The description of the setting is thin and insufficient to clarify the policy context. I suggest the authors provide more details and explanation about the “Treat All” policy and its implementation in Kenya – when, what, who, and how.**

We have added the following text to the Setting section:

“Kenya has one of the largest HIV burdens in the world with an HIV prevalence of 6.1% among women 15-49 years of age [32]. Kenya adopted the Option B+ policy in 2014, and in 2016, this policy was subsumed by the WHO “Treat all” policy (i.e., universal test-and-treat strategy for all persons with HIV after diagnosis regardless of age, CD4 count, and pregnancy status) [33, 34]. In addition, routine HIV services were integrated within public maternal and child health clinics in 2015. In this integrated model, HIV services are delivered in antenatal clinics (ANC) and postnatal clinics (PNC) rather than in HIV clinics. These policies have facilitated a significant scale-up of HIV testing and antiretroviral treatment (ART) delivery for general populations and PPHIV in Kenya [35]. As of 2020, an estimated 85% of pregnant women presenting at ANC in Kenya were tested for HIV or knew their HIV status, and 94% of pregnant women living with HIV accessed antiretroviral therapy (ART) for PMTCT [32]. The scale-up of ART has also helped to drive significant reductions in vertical transmission in Kenya, from 26% in 2009 to 11% in 2018 [36-38].”

3. **Why were 41 participants interviewed? This is a reasonable sample size, but I wasn’t sure how you reached that number. Did you sample until data saturation? Or was the sampling size determined by time and resource constraints?**

We have added the following text to the Methods:

“We aimed to sample at least 40 PPHIV, including at least 15 LTFU women, for the interviews. This sample size was estimated to be appropriate for achieving data saturation based on our knowledge of the related literature and our prior qualitative research experience in Kenya, as well as the time and resource constraints of the study.”

4. **The conceptual framework is sound. I particularly liked the way in which the four domains are used (ie individual, family, community, health system level) to organise the presentation of findings. It is also good you recognized that some themes such as stigma are cross cutting. However, I wonder how the framework in Figure 1 was used in the research process. Was it developed to guide data collection, data analysis, or both? I am also not so sure Figure 1 is really necessary. Perhaps that figure could be refined in light of the findings (and placed in the result section or at the beginning of the discussion).**

We used this conceptual framework to guide data collection, analysis and to organize the write-up of our findings. To clarify this, we have included additional details under the “Conceptual framework” section as follows:

“We developed a conceptual framework based on the Social-Ecological Model and used it across the research process, including to drive data collection and the development of the interview guide and the analysis (Fig 1) [24, 31, 43, 44].”

We have also included additional details under the “Data collection and management” section as follows:

“After adapting the Social-Ecological Model to PMTCT in the Kenyan context, we used it to guide the development of an interview protocol that consisted of semi-structured and open-ended questions. This ensured that we were exploring the central topics within each domain in the model and identified in the literature, while still allowing for flexibility and room for women to discuss and add their own perceived and experienced barriers and enhancers to retention in care for PPHIV and their infants.”

Regarding Figure 1, we would prefer that it be introduced in the Methods section rather than elsewhere in the manuscript in order to illustrate the adapted conceptual framework we used during the study, and serve as a visual aide for the reader to help set up the presentation of the results.

5. **In the methods section, the authors mention that "new codes were developed inductively using axial and selective coding". However, there is no reference to open coding (which is the foundation of inductive analysis).**

We have added detail to the analysis section to clarify and more thoroughly describe the coding process as follows:

“Patient characteristics at enrollment were summarized descriptively using Stata version 10.3. For the analysis, four investigators (J.H., B.K., M.A. and J.W.) began with an open coding framework to identify themes emerging from the data and across interviews. Once a theme was provisionally defined, additional examples were sought during subsequent readings. To ensure validity and reliability, inductive codes were discussed among three investigators (J.H., B.K, and M.A.) to establish a consensus prior to adding them to the codebook. These codes were refined and new codes developed inductively using axial and selective coding of transcripts during the analysis and were informed by our Social-Ecological Model, literature review, and additional themes arising during interviews. Axial coding of each transcript was performed independently by two investigators (J.H. and M.A.), using analytic memos and decision trails to ensure coding consistency and resolving differences through discussion with a third investigator (B.K.). Nvivo 12 was used for the qualitative analysis. Illustrative quotes were selected to highlight key themes that emerged across the data. Data from structured questionnaires and interviews were triangulated to further enhance the construct validity and trustworthiness of the inferences.”

6. **The section on “mobility” should be revised. In particular, the following statement needs explanation: “the cultural tradition in which women traveled to their mothers' home during pregnancy or the postpartum period, was another potential barrier to retention”.**

We have revised the section on mobility as follows (see underlined text):

“Mobility, in part related to the cultural tradition in which women traveled to their mothers’ home during pregnancy or the postpartum period to receive care from parents or other family members, was another potential barrier to retention. This has been described in Kenya [46] and South Africa [3], and may be influenced by the nature of the study sites as referral facilities with sizeable catchment areas, where some women may prefer to seek longitudinal care at lower-level services closer to their family.”

7. **The conclusion in the abstract could be more forceful**

We have edited the conclusion by removing equivocal language (e.g., ‘may also’, ‘are likely’) as follows:

“PPHIV must navigate multiple, sometimes conflicting social roles, statuses and identities related to HIV, motherhood, and social class, to maintain retention in care. Interventions to address multiple barriers simultaneously will have the greatest impact given the complex nature of retention for this population. Such interventions will need to be highly contextualized and individualized.”

8. **HIV is ok in the title but less known acronyms such as EA-IeDEA and PMTCT should not be used**

We have removed the acronyms such that the title is now “A qualitative study of the barriers and enhancers to retention in care for pregnant and postpartum women living with HIV”. We have also removed the use of “EA-IeDEA” as an abbreviated study title in the manuscript text.
